# CC16 polymorphisms in asthma, asthma subtypes, and asthma control in adults from the Agricultural Lung Health Study

**DOI:** 10.1186/s12931-022-02211-6

**Published:** 2022-11-09

**Authors:** KC Gribben, AB Wyss, JA Poole, PA Farazi, C Wichman, M Richards-Barber, LE Beane Freeman, PK Henneberger, DM Umbach, SJ London, TD LeVan, Kelli C. Gribben

**Affiliations:** 1 Department of Epidemiology, University of Nebraska Medical Center, 68198 Omaha, NE USA; 2 Epidemiology Branch, National Institute of Environmental Health Sciences, Department of Health and Human Services, National Institutes of Health, Research Triangle Park, NC USA; 3 Department of Internal Medicine, Division of Allergy and Immunology, University of Nebraska Medical Center, 68198 Omaha, NE USA; 4 Department of Biostatistics, University of Nebraska Medical Center, 68198 Omaha, NE USA; 5 Westat, Durham, NC USA; 6 Biostatistics and Computational Biology Branch, National Institute of Environmental Health Sciences, Department of Health and Human Services, National Institutes of Health, Research Triangle Park, NC USA; 7 Occupational and Environmental Epidemiology Branch, National Cancer Institute, Bethesda, MD USA; 8 Respiratory Health Division, National Institute for Occupational Safety and Health, Centers for Disease Control and Prevention, Morgantown, WV USA; 9 Department of Internal Medicine, Division of Pulmonary, Critical Care and Sleep, University of Nebraska Medical Center, 68198 Omaha, NE USA

**Keywords:** CC16 polymorphisms, Adult asthma, Asthma control

## Abstract

**Background:**

The club cell secretory protein (CC16) has anti-inflammatory and antioxidant effects and is a potential early biomarker of lung damage. The *CC16* single nucleotide polymorphism (SNP) rs3741240 risk allele (A) has been inconsistently linked to asthma; other tagging SNPs in the gene have not been explored. The aim was to determine whether *CC16* tagging polymorphisms are associated with adult asthma, asthma subtypes or asthma control in the Agricultural Lung Health Study (ALHS).

**Methods:**

The ALHS is an asthma case-control study nested in the Agricultural Health Study cohort. Asthma cases were individuals with current doctor diagnosed asthma, likely undiagnosed asthma, or asthma-COPD overlap defined by questionnaire. We also examined asthma subtypes and asthma control. Five *CC16* tagging SNPs were imputed to 1000 Genomes Integrated phase 1 reference panel. Logistic regression was used to estimate associations between *CC16* SNPs and asthma outcomes adjusted for covariates.

**Results:**

The sample included 1120 asthma cases and 1926 controls of European ancestry, with a mean age of 63 years. The frequency of the risk genotype (AA) for rs3741240 was 12.5% (n = 382). *CC16* rs3741240 was not associated with adult asthma outcomes. A tagging SNP in the CC16 gene, rs12270961 was associated with uncontrolled asthma (n = 208, OR_adj_= 1.4, 95% CI 1.0, 1.9; p = 0.03).

**Conclusion:**

This study, the largest study to investigate associations between *CC16* tagging SNPs and asthma phenotypes in adults, did not confirm an association of rs3741240 with adult asthma. A tagging SNP in CC16 suggests a potential relationship with asthma control.

**Supplementary Information:**

The online version contains supplementary material available at 10.1186/s12931-022-02211-6.

## Background

Asthma is a chronic inflammatory airway disease of complex etiology with a significant genetic contribution estimated to range between 35-70% [1, 2]. Environmental factors also play a critical role in asthma development and severity, as evidenced by the dramatic increasing trend in prevalence between 1980-2010 [3, 4] Genes and associated variants may indirectly affect susceptibility to asthma by altering normal processes in the lung in response to environmental agents [5–8].

The club cell secretory protein (CC16) has been implicated in multiple pulmonary diseases, including asthma, chronic obstructive pulmonary disease (COPD), and smoking-induced lung cancer [9–13]. The *CC16* gene found on chromosome 11q12.3, is highly expressed in the lung, and encodes a seemingly beneficial pneumoprotein, CC16 [14]. Abundantly secreted by non-ciliated club cells, [14] CC16 exerts anti-inflammatory and antioxidant effects [10, 14] and moves across the lung epithelial-blood barrier upon inhalation of environmental respiratory insults [10, 15−19]. In healthy adults, the correspondence between CC16 levels in serum and in bronchoalveolar lavage (BAL) fluid suggests its potential use as an early biomarker of lung injury and early development of respiratory disease [15]. Low serum CC16 serum levels have been associated with asthma, airway hyper-responsiveness, COPD severity, and lung function [11, 20, 21]. *CC16* polymorphisms also influence CC16 protein levels [22, 23]. Laing et al., reported differences in plasma CC16 levels by the rs374120 genotype, with lowest levels seen in children homozygous for the A allele (CC16 concentrations by genotype: AA 6.79 µg/L, AG 9.17 µg/L and GG 10.01 µg/L) [9]. The influence of rs374120 genotype on CC16 levels continues into adulthood [22] with *CC16* polymorphisms (including rs374120) estimated to account for 6% of the variability in circulating CC16 levels [24].

Genetic association studies of *CC16* polymorphisms have been inconsistently associated with asthma in child and adult populations [25–27]. One study estimated children with the risk genotype (AA) for rs3741240 had nearly four times greater odds of asthma than those with the GG genotype [26]. Additionally, a recent meta-analysis analyzed data from 19 case-control studies and reported that those with the AA or AG genotype for *CC16* rs3741240 had 1.3 times greater odds of asthma compared to the GG genotype (Pooled Odds Ratio = 1.29; 95% CI 1.08, 1.54) [27]. However, this effect was attenuated once studies contributing to heterogeneity, identified using Galbraith plots, were excluded [27]. All studies in the meta analysis had fewer than 650 asthmatics and fewer than 85 with the risk genotype [27]. These inconsistent results are not surprising given that asthma is a heterogeneous disease, the meta-analysis included studies from around the world with likely varying environmental exposures, and allele frequencies of the polymorphism vary by ethnicity.

The farming environment has historically been of interest to allergy and asthma researchers due to the unique diversity of exposures to bacteria, fungi, chemicals, and allergens that may influence risk [28–32]. Compared to the general population, farmers experience a lower prevalence of asthma diagnosis but more respiratory symptoms, including wheeze, cough, and phlegm [33]. Early-life and current farming exposures have been inconsistently associated with atopy or asthma in adults [33–44]. Among previous genetic association studies of *CC16* polymorphisms and asthma, none were conducted within an agricultural population. Furthermore, many of these studies had modest sample sizes, were not limited to adults, were conducted outside the United States, and lacked defined asthma subtypes such as atopy, eosinophilia, asthma control, and exacerbations. No other polymorphisms in *CC16* besides rs3741240 have been studied. Therefore, this study aims to determine whether *CC16* tagging polymorphisms are associated with adult asthma, asthma subtypes, and asthma control, including exacerbations in adults, from the Agricultural Lung Health Study (ALHS). We also examined associations of *CC16* polymorphisms with wheeze and with chronic bronchitis.

## Methods

The Agricultural Lung Health Study (ALHS), a nested case-control study of adult asthma drawn from the larger Agricultural Health Study (AHS) [36, 45], enrolled 3301 individuals including 1223 asthma cases and 2078 controls [36]. The sample is predominantly farmers and farm spouses from Iowa and North Carolina [36, 45].

### CC16 polymorphisms

Polymorphisms in the *CC16* gene were genotyped using the UK Biobank Axiom Array by Affymetrix Asiom Genotyping Services with DNA extracted from blood or saliva from ALHS population [46]. Genetic variants were imputed to 1000 Genomes Integrated phase 1 reference panel [46].

For this study, we analyzed individuals of European ancestry (n = 3046 including 1,120 individuals with asthma) based on genome-wide association study (GWAS) ancestral principal components. We identified five tagging polymorphisms in the *CC16* (SCGB1A1) gene: rs3741240, rs4963506, rs12270961, rs10897271, and rs11231085 using Genome Variation Server, HapMap and Complete Genomics with an r^2^ cutoff of 0.7 [47]. Allele frequencies for rs10897271 and rs11231085 were not in Hardy-Weinberg Equilibrium, leaving three tagging polymorphisms for the analysis (i.e. rs3741240, rs4963506, rs12270961) [47].

### Asthma

Current asthma cases were identified from responses to the most recent follow-up questionnaire for the parent AHS cohort [36]. The majority (n = 876) of cases self-reported current asthma by responding “yes” to the two questions “have you ever been diagnosed with asthma?” and “do you still have asthma?” and “no” to the two questions “have you ever been diagnosed with chronic obstructive pulmonary disease (COPD)?” and “have you ever been diagnosed with emphysema?”. To avoid missing undiagnosed asthma, 309 participants were classified as undiagnosed current asthma if they denied having a previous diagnosis of asthma, COPD, or emphysema but reported current asthma symptoms and the use of asthma medications, and a history of never smoking (n = 263) or minimal past smoking (≤ 10 pack-years, n = 46). Because of possible asthma and COPD overlap, we included 38 individuals as asthma cases based on reporting current asthma and a previous diagnosis of either COPD or emphysema as long as they were never-smokers (n = 28) or past-smokers (≤ 10 pack-years, n = 10) [36]. Controls were randomly selected from participants not meeting the asthma case definitions at the time of enrollment [36].

### Asthma subtypes

Atopy was defined by having at least one of ten individual allergen-specific immunoglobulin E (IgE) ≥ 0.70 IU/mL [36]. Allergens included seasonal (Bermuda grass, ragweed, timothy grass, mountain cedar), perennial (Alternaria, dust mite, cat dander), and food (milk, egg, wheat). Atopic asthma subtypes were categorized into four groups: atopic asthma, non-atopic asthma, atopy alone, no atopy & no asthma. The eosinophilic asthma subgroups were defined using fractional exhaled nitric oxide (FeNO) > 50 ppb for eosinophilic asthma, non-eosinophilic asthma ≤ 50 ppb, eosinophilic alone > 50 ppb, and no asthma and not eosinophilic [48]. A sensitivity analysis was conducted classifying asthmatics as low FeNO < 25 ppb, intermediate FeNO 25–50 ppb, and high FeNO > 50 ppb.

### Asthma Control and Exacerbations

Among adults diagnosed with asthma, asthma control was defined using the validated Asthma Control Questionnaire (ACQ), with the modification that questions referenced the past two weeks instead of the past week [49]. For each individual, each question is scored ranging from 0 (no impairment) to 6 (maximum impairment). Pre-bronchodilator FEV_1_ percent predicted is also scored 0 to 6. The final score is the mean of the seven items (questionnaire and lung function) which ranges from 0 (totally controlled) to 6 (severely uncontrolled) [49, 50]. A binary variable, controlled (ACQ score < 1.5) versus uncontrolled (ACQ score ≥ 1.5) asthma was defined according to clinical guidelines from Global Initiative for Asthma (GINA) [51]. A sensitivity analysis was conducted with asthma control as 3-levels (well controlled 0-0.75, moderately controlled 0.76–1.5, uncontrolled > 1.5) [51]. Asthma control was only evaluated among cases that self-reported current asthma [50]. Among adults with diagnosed asthma, asthma exacerbations were defined as ‘Yes’ to at least one of two questions “In the past 12 months did you get urgent treatment for an asthma attack at a doctor’s office, urgent care facility, or ER?” or “In the past 12 months have you been hospitalized overnight for asthma?”

### Respiratory symptoms

Wheeze was defined as having wheezing or whistling in your chest during the past 12 months or wheeze when exerting during the past 12 months. Chronic cough was defined as having a cough on most days, for three consecutive months or more during the year for a duration of two years or more. Similarly, chronic phlegm was defined as bringing up phlegm on most days for three consecutive months or more during the year, for a duration of two years or more. Chronic bronchitis was defined as individuals self-reporting both chronic cough and phlegm.

### Statistical analysis

We summarized demographic and clinical characteristics overall and by asthma status using basic descriptive statistics and evaluated univariate associations of *CC16* polymorphisms with each respiratory outcome and each covariate using t-tests for continuous variables and Chi-square tests for categorical variables. Multinomial logistic regression models with generalized logit were used to evaluate associations between *CC16* polymorphisms and asthma subtypes. Logistic regression models were used to evaluate associations between binary outcomes in analyses limited to diagnosed asthmatics, asthma control (controlled/uncontrolled), and having an exacerbation in the past year (yes/no). We assumed an additive genetic effect of *CC16* polymorphisms on respiratory outcomes. With rs3741240 genotypes coded as AA = 2, AG = 1, GG = 0 and rs12270961 genotypes coded as GG = 2, AG = 1, AA = 0 and analyzed as ordinal variables. In sensitivity analysis, genotypes were analyzed as a 3-level categorical variable. For rs3741240 categorical genotype AA, AG, GG (reference) and for rs12270961 GG, AG, and AA (reference) based on published evidence of risk alleles [9, 27].

All regression models were adjusted for age (continuous), sex (Female or Male), state (Iowa or North Carolina), and smoking status (current, former, never). Additionally, asthma control and exacerbation models were adjusted for atopy; wheeze and chronic bronchitis models were adjusted for asthma status. Goodness of fit was evaluated using the Hosmer-Lemeshow test. Sensitivity analyses were conducted to ensure the robustness of results and are described in the results section. The predetermined level of significance was two sided alpha < 0.05 and SAS (Cary, NC) was used for all analyses. Linkage Disequilibrium plot was created in Haploview version 4.2 [52].

## Results

The present analysis included 3046 farmer and spouse participants from the ALHS with information on *CC16* polymorphisms. Participants had an average age of 63 years, and most were current farmers (63%), and never (65%) or former (29%) smokers. Of the 1,120 adults with asthma, the majority were non-atopic (71%) and non-eosinophilic (87%) (Table 1).

**Table 1:** Characteristics of the Agricultural Lung Health Study Population

	Total n = 3046	Asthma n = 1120	No Asthma n = 1926
**Age**, mean (SD)	62.6 (11.1)	61.8 (10.8)	63.1 (11.2)
**Gender**, n (%)			
Male	1566 (51.4)	528 (47.1)	888 (46.1)
Female	1480 (48.6)	592 (52.9)	1038 (53.9)
**BMI** kg/m^2^, mean (SD)	30.15 (6.1)	31.32 (6.7)	29.50 (5.6)
Missing	40 (1.3)	18 (1.6)	22 (1.1)
**State**, n (%)			
Iowa	2179 (71.5)	805 (71.9)	1374 (71.3)
North Carolina	867 (28.5)	315 (28.1)	552 (28.7)
**Race**, n (%)			
White	3043 (99.9)	1120 (100.0)	1923 (99.8)
Other	3 (0.1)	0 (0.0)	3 (0.2)
**Smoking status**, n (%)			
Never	1982 (65.1)	765 (68.3)	1217 (63.2)
Former	890 (29.2)	308 (27.5)	582 (30.2)
Current	126 (4.1)	32 (2.9)	94 (4.9)
Missing	48 (1.6)	15 (1.3)	33 (1.7)
Pack-years*, median (IQR)	9.0 (25.5)	6.5 (18.5)	11.3 (28.5)
Missing	4 (0.1)	3 (0.3)	1 (0.03)
**Currently Farming**^**†**^, n (%)	1908 (62.6)	688 (61.4)	1220 (63.3)
Missing	1 (0.03)	1 (0.03)	0 (0.0)
**Family history of asthma**, n (%)	807 (26.5)	449 (40.1)	358 (18.6)
Missing	34 (1.1)	16 (1.4)	18 (0.9)
**Atopy**^**‡**^, n (%)			
Yes	543 (17.8)	299 (26.7)	244 (12.7)
No	2428 (79.7)	795 (71.0)	1633 (84.8)
Missing	75 (2.5)	26 (2.3)	49 (2.5)
**FeNO**^**§**^, n (%)			
≤ 50 ppb	2746 (90.2)	971 (86.7)	1775 (92.2)
>50 ppb	96 (3.2)	68 (6.1)	28 (1.5)
Missing	204 (6.7)	81 (7.2)	123 (6.4)
**Asthma Control among diagnosed asthmatics**^**||**^, n (%)			
Controlled		522 (46.6)	-
Uncontrolled		208 (18.6)	-
Missing		390 (34.8)	-
**Exacerbations past 12 months**^**¶**^, n (%)			
Yes		59 (5.3)	-
No		1015 (90.6)	-
Missing		46 (4.1)	-
**rs3741240**			
AA	382 (12.5)	133 (11.9)	249 (12.9)
AG	1344 (44.1)	501 (44.7)	843 (43.8)
GG	1320 (43.3)	486 (43.4)	834 (43.3)
HWE p-value	0.17	0.82	0.12
**rs4963506**			
AA	119 (3.9)	43 (3.8)	76 (4.0)
AG	999 (32.8)	367 (32.8)	632 (32.8)
GG	1928 (63.3)	710 (63.4)	1218 (63.2)
HWE p-value	0.46	0.60	0.59
**rs12270961**			
AA	121 (4.0)	44 (3.9)	77 (4.0)
AG	1009 (33.1)	370 (33.0)	639 (33.2)
GG	1916 (62.9)	706 (63.0)	1210 (62.8)
HWE p-value	0.41	0.60	0.52

Definition of abbreviation: HWE = Hardy-Weinberg Equilibrium

*Pack-years among former or current smokers

†Currently farming defined as during the past 12 months spent time growing, harvesting crops or any farm work related to growing or harvesting soybeans, wheat, corn, hay, straw, tobacco, cotton or other crops and/or worked with farm animals

‡Atopy defined as allergen-specific IgE to at least one of 10 allergens using a threshold of ≥ 0.70 IU/ml. Seasonal: Bermuda grass, ragweed, timothy grass, mountain cedar; Perennial: Alternaria, dust mite, cat dander; Food: milk, egg, and wheat

§Fractional exhaled nitric oxide (FeNO) > 50 ppb used to define eosinophilic asthma subtype, ≤ 50 ppb used to define non-eosinophilic asthma subtype

││Asthma control: controlled < 1.5 score, uncontrolled ≥ 1.5 score in people with diagnosed current asthma or ACO (n = 829). The majority of adults identified as undiagnosed asthma did not receive questions about asthma control

¶Exacerbation defined as ‘Yes’ to at least one of two questions “In the past 12 months did you get urgent treatment for an asthma attack at a doctor’s office, urgent care facility, or ER?” or “In the past 12 months have you been hospitalized overnight for asthma?” among people with asthma

**Fig. 1:**
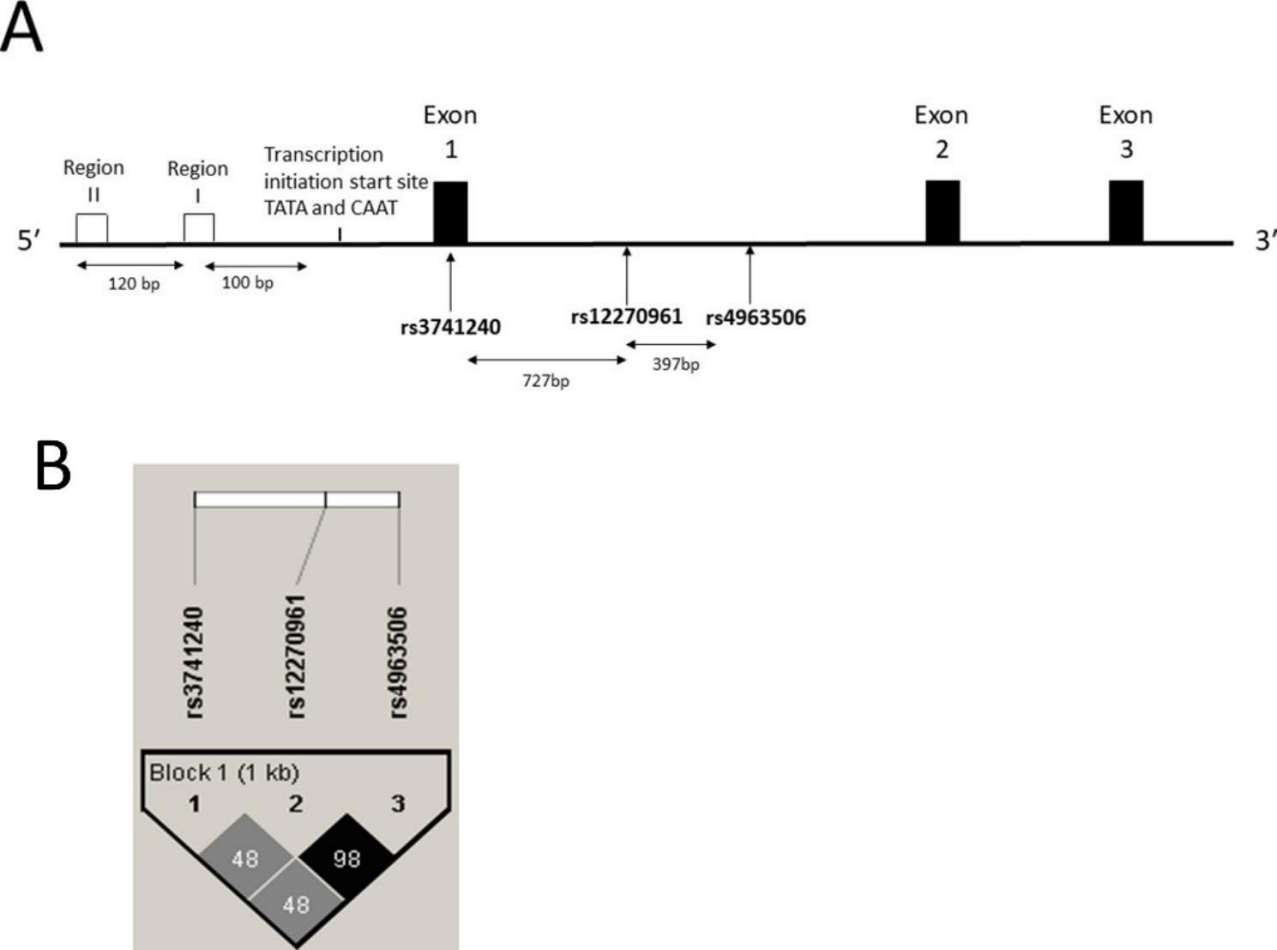
A. Diagram of the *CC16* (SCGB1A1) gene. In humans, the *CC16* gene is located on chromosome 11q12.3. The gene is 18,108 bp in length, includes three exons and two introns. The rs3741240 polymorphism is in the untranslated region of Exon 1 which is downstream from the transcription initiation site. rs12270961 and rs4963506 are located 730 bp downstream within intron 1. B. Linkage disequilibrium (R2) plot for *CC16* polymorphisms. rs12270961 and rs4963506 are in nearly complete linkage

The location of the tagging *CC16* polymorphisms, linkage, and frequencies including Hardy-Weinberg Equilibrium (HWE) are displayed in Fig. 1 and Table 1. In the ALHS, the frequency of the AA genotype for the rs3741240 polymorphism was 12.5%. The tagging polymorphisms rs4963506 and rs12270961 are in complete linkage disequilibrium. Therefore, we have chosen to present study results based on rs12270961 since neither SNP has been described in the published literature. The frequency of genotypes AA, AG, GG for rs12270961 this variant were 4%, 33%, and 63%, respectively. Genotype frequencies for the two polymorphisms (rs3741240 and rs12270961) did not vary by asthma status (Table 1).

*CC16* polymorphisms (rs3741240 and rs12270961) were not associated with asthma, asthma subtypes (Table 2), chronic bronchitis, or wheeze (Table 4). In sensitivity analyses of chronic bronchitis or wheeze, we performed a stratified analysis by asthma status, but results and conclusions were unchanged (Supplemental Tables 2 and 3). An additional sensitivity analysis was conducted examining relationships between asthma outcomes and *CC16* rs3741240 treating the genotype as a 3-level categorical variable and there was no association (Supplemental Table 1). We also performed a sensitivity analysis with the outcome child-onset asthma, defined as asthma diagnosed on or before age 17 versus after age 17, and results were largely similar to the findings for asthma and asthma subtypes: rs3741240 Odds Ratio = 1.0 (95% CI 0.8, 1.2), p = 0.66), rs12270961 Odds Ratio = 1.1 (95% CI 0.9, 1.4), p = 0.45). For the eosinophilic asthma subtype outcome, we ran an additional model combining the AA and AG genotypes for rs12270961 (GG was reference) and results were not statistically significant (p = 0.93). Lastly, when we used a common atopy cutpoint of 0.35 (instead of 0.70) and re-analyzed the atopic asthma subtype outcome, results were broadly similar (Supplemental Table 4).

**Table 2:** Associations of CC16 polymorphisms with asthma and asthma subtypes in the ALHS (N = 3046)

	CC16 rs12270961					CC16 rs3741240				
	**Genotype n (row %)**					**Genotype n (row %)**				
**Outcome**	**AA**	**AG**	**GG**	**Adj OR** (95% CI)*	**p**	**AA**	**AG**	**GG**	**Adj OR** (95% CI)*	**p**
**Asthma**					0.98					0.69
No	77 (4.0)	639 (33.2)	1210 (62.8)	**Ref**		249 (12.9)	843 (43.8)	834 (43.3)	**Ref**	
Yes	44 (3.9)	370 (33.0)	706 (63.0)	1.0 (0.9,1.1)		133 (11.9)	501 (44.7)	486 (43.4)	1.0 (0.9,1.1)	
**Atopy/Asthma** ^*^					0.83					0.78
Neither	63 (3.9)	541 (33.1)	1029 (63.0)	**Ref**		209 (12.8)	718 (44.0)	706 (43.2)	**Ref**	
Atopy only	14 (5.7)	75 (30.7)	155 (63.5)	1.0 (0.8,1.2)		33 (13.5)	100 (41.0)	111 (45.5)	1.0 (0.8,1.2)	
Asthma only	33 (4.2)	264 (33.2)	498 (62.6)	1.0 (0.8,1.1)		102 (12.8)	352 (44.3)	341 (42.9)	1.0 (0.9,1.1)	
Both	10 (3.3)	95 (31.8)	194 (64.9)	1.1 (0.9,1.3)		30 (10.0)	137 (45.8)	132 (44.2)	0.9 (0.8,1.1)	
**Eosinophilia/Asthma** ^†^					0.94					0.96
Neither	69 (3.9)	587 (33.1)	1119 (63.0)	**Ref**		229 (12.9)	765 (43.1)	781 (44.0)	**Ref**	
Eosinophilia only	1 (3.6)	9 (32.1)	18 (64.3)	1.2 (0.6,2.5)		4 (14.3)	14 (50.0)	10 (35.7)	1.2 (0.7,2.0)	
Asthma only	39 (4.0)	319 (32.9)	613 (63.1)	1.0 (0.9,1.1)		117 (12.1)	438 (45.1)	416 (42.8)	1.0 (0.9,1.1)	
Both	3 (4.4)	23 (33.8)	42 (61.8)	0.9 (0.6,1.4)		11 (16.2)	26 (38.2)	31 (45.6)	1.0 (0.7,1.5)	

Additive genetic model for the rs12270961 genotype coded as the number of copies of the G allele; for the rs3741240 genotype, as the number of copies of the A allele; thus, ORs reflect a multiplicative change in odds per additional copy of the designated allele

Multivariable logistic regression model to estimate Odds Ratios and 95% confidence intervals. Asthma model adjusted for gender, state (NC, IA), age, smoking status (current, former, never). Multinomial logistic regression model to estimate adjusted odds ratios and 95% confidence intervals for each asthma subtype outcome category compared to reference category per each additional allele. Adjusted for gender, state (NC, IA), age, smoking status (current, former, never). Models were not adjusted for race because sample population is 99.9% white

*Atopy/asthma = atopy defined as 1 or more allergen-specific IgE ≥ 0.70 IU/mL cross-classified with self-reported asthma (yes/no) resulting in four groups

†Eosinophilia/asthma = eosinophilic defined by fractional exhaled nitric oxide (FeNo) > 50 ppb cross-classified with self-reported asthma (yes/no) resulting in four groups

Among adults with asthma, assuming an additive genetic model, rs12270961 was associated with uncontrolled asthma (Table 3). Each additional copy of the G-allele, was associated with a 1.4-fold increase in the odds of uncontrolled asthma (95% CI 1.02,1.91; p = 0.03). If we do not assume an additive genetic model, the odds ratios are 0.7 (95% CI 0.3,1.8) for the AG genotype and 1.2 (95% CI 0.5,2.8) for the GG genotype compared to AA genotype.

**Table 3:** Associations between CC16 polymorphisms and uncontrolled asthma and exacerbations in the ALHS (N = 1120)

	CC16 rs12270961					CC16 rs3741240				
	**Genotype n (row %)**					**Genotype n (row %)**				
**Outcome**	**AA**	**AG**	**GG**	**Adj OR** (95% CI)*	**p**	**AA**	**AG**	**GG**	**Adj OR** (95% CI)*	**p**
**Asthma Control** ^*^					0.03					0.64
Controlled	22 (4.2)	189 (36.2)	311 (59.6)	**Ref**		70 (13.4)	230 (44.1)	222 (42.5)	**Ref**	
Uncontrolled	8 (3.9)	58 (27.9)	142 (68.3)	1.4 (1.0, 1.9)		25 (12.0)	90 (43.3)	93 (44.7)	0.9 (0.7, 1.2)	
**Asthma exacerbation past 12 months** ^†^					0.40					0.16
No	41 (4.0)	335 (33.0)	639 (63.0)	**Ref**		125 (12.3)	451 (44.4)	439 (43.3)	**Ref**	
Yes	2 (3.4)	16 (27.1)	41 (69.5)	1.2 (0.8,2.1)		4 (6.8)	25 (43.4)	30 (50.9)	0.7 (0.5, 1.1)	

Additive genetic model for the rs12270961 genotype coded as the number of copies of the G allele; for the rs3741240 genotype, as the number of copies of the A allele; thus, ORs reflect a multiplicative change in odds per additional copy of the designated allele

*Asthma control: controlled < 1.5 score, uncontrolled ≥ 1.5 score. 730 adults with asthma had asthma control data. Multivariable logistic regression model was fit with 722 observations (8 subjects lost due to missing a value for atopy) and adjusted for age, gender, state (IA or NC), smoking status (never, former, current), and atopy

†Exacerbation defined as ‘Yes’ to at least one of two questions: “In the past 12 months did you get urgent treatment for an asthma attack at a doctor’s office, urgent care facility, or ER?” or “In the past 12 months have you been hospitalized overnight for asthma?”

Multivariable logistic regression model adjusted for age, gender, state (IA or NC), smoking status (never, former, current), and atopy

**Table 4:** Associations between CC16 polymorphisms and chronic bronchitis and wheeze in the ALHS (N = 3046)

	CC16 rs12270961					CC16 rs3741240				
	**Genotype n (row %)**					**Genotype n (row %)**				
**Outcome**	**AA**	**AG**	**GG**	**Adj OR** (95% CI)*	**p**	**AA**	**AG**	**GG**	**Adj OR** (95% CI)*	**p**
Chronic Bronchitis*					0.85					0.98
No	108 (4.0)	899 (33.2)	1705 (62.9)	**Ref**		345 (12.7)	1193 (44.0)	1174 (43.3)	**Ref**	
Yes	12 (4.2)	91 (32.0)	181 (63.7)	1.0 (0.8,1.3)		34 (12.0)	126 (44.4)	124 (43.7)	1.0 (0.8,1.2)	
**Wheeze** ^†^					0.56					0.25
No	79 (4.0)	652 (32.7)	1265 (63.4)	**Ref**		260 (13.0)	851 (42.64)	885 (44.3)	**Ref**	
Yes	41 (4.1)	337 (33.8)	620 (62.1)	1.0 (0.8,1.1)		119 (11.9)	467 (46.8)	412 (41.3)	1.1 (0.9,1.2)	

Additive genetic model for the rs12270961 genotype coded as the dnumber of copies of the G allele; for the rs3741240 genotype, as the number of copies of the A allele; thus, ORs reflect a multiplicative change in odds per additional copy of the designated alleleMultivariable logistic regression model adjusted for age, gender, state, smoking status, and asthma status

*Chronic bronchitis defined as individuals with both chronic cough and phlegm. Chronic cough defined as having a cough on most days, for three consecutive months or more during the year for a duration of two years or more. Similarly, chronic phlegm defined as bringing up phlegm on most days for three consecutive months or more during the year, for a duration of two years or more

†Wheeze defined as having wheezing or whistling in your chest during the past 12 months or wheeze when exerting during the past 12 months

A small number of participants with asthma experienced an exacerbation defined as an urgent healthcare visit for an asthma attack or overnight hospitalization for asthma in the past 12 months (2%, n = 59) and the *CC16* polymorphisms were not associated with asthma exacerbations (Table 3).

## Discussion

This is the first study to investigate the genetic effects of *CC16* polymorphisms on adult asthma in a large United States sample. We did not find evidence of an association between *CC16* polymorphisms with adult asthma, asthma subtypes, or respiratory symptoms. Our findings are consistent with several published studies, many of which were included in a meta-analyses by Nie et al. (19 case-control or cohort studies) and Cheng et al. (15 case-control studies), which both concluded *CC16* rs3741240 was not associated with an increased risk of asthma [27, 53]. Several individual studies noted no association between *CC16* rs3741240 and asthma in adults but there were several methodological differences that limit comparisons to the present study. For example, there were differences in how asthma was defined, inclusion/exclusion criteria, study location, and age of study population [54–58]. A limited number of studies have shown an increased odds of asthma in adults carrying the risk allele for rs3741240 [25, 27]. In a study by Taniguchi et al., they did not observe an association with asthma overall in Japanese adults but did report an association with late-onset asthma, diagnosed after age 40 (OR_adj_ = 1.62 95% CI 1.09–2.40) adjusted for age, sex, atopy, and smoking status [25]. Differences in the classification of asthma, risk genotype frequency (higher in Japanese 18.8% vs. Europeans 11–12%) and environmental factors likely contributed to differences between their findings and ours. However, a novel association between a *CC16* tagging SNP, rs12270961, and uncontrolled asthma was observed in this study.

Since asthma is a heterogenous disease, in addition to asthma overall, we investigated relationships between *CC16* polymorphisms with asthma subtypes. We demonstrated no relationships between *CC16* polymorphisms with asthma subtypes, atopic asthma, or eosinophilic asthma. Consistent with our findings, other studies have failed to observe an association between *CC16* variants and atopy, atopic asthma, or eosinophilic inflammation. Nie et al. pooled data from all ages and found no evidence of a relationship between *CC16* rs3741240 polymorphism with atopic asthma (OR = 1.1, 95% CI = 0.8–1.5, p = 0.57) [27]. It is worth noting, though, that atopic status was defined differently (i.e. skin prick test, total IgE, specific IgE) across the individual studies included in the meta-analysis [27]. Most commonly, atopy was included as a covariate in an asthma model and previous studies did not stratify asthma by atopic status [25, 54, 56, 57, 59]. Furthermore, Mansur et al. did not find an association between *CC16* rs3741240 and adult asthma, the majority (86%) of which were atopic based on skin prick test to common allergens [54]. Others included atopy as a covariate in the analysis, not an outcome, or only presented unadjusted or crude results [25, 58]. Candelaria and colleagues noted an increased odds of atopic dermatitis (adjusted for age and gender) in an adult Danish population associated with the A-allele for rs3741240; no association was found with rhinitis or atopy, nor an interaction between current asthma*atopy [57]. Conversely, in atopic children with allergic rhinitis (mean age 11 years), Ku et al. noted an increased odds (unadjusted) of asthma in children with AA genotype compared to GG + AG [60].

Evaluation of genetic associations between *CC16* polymorphisms and eosinophilic asthma in adults using FeNO, a biomarker of “allergic” inflammation, yielded no significant findings in our study. A study in children demonstrated that the AA genotype for rs3741240 was associated with elevated FeNO (> 30 ppb) in girls (not boys), but the sample size was minimal, with only six girls having the risk genotype of interest [61]. Other studies that measured blood eosinophils, but did not define asthma subtypes, found no association with *CC16* rs3741240 [57, 60]. Unlike these previous studies, our sample of adults were primarily non-atopic and non-eosinophilic, which is consistent with existing knowledge of adult farming populations. Farmers often have a lower prevalence of allergic diseases and inflammatory markers (IgE, eosinophils) resulting from agricultural environmental exposures [28]. Therefore, our study contributes new information on *CC16* polymorphisms in adults with non-allergy-driven asthma. While it does not appear the *CC16* rs3741240 has a role in adult atopic or non-atopic asthma, our findings need to be confirmed in independent study populations.

We did find an association between *CC16* polymorphisms and asthma control in ALHS. Asthma control is an indicator of how well the disease is being managed, and changes in lung function or symptoms (frequency or severity) over time may guide clinical decisions. Similarly, exacerbations can also inform treatment and management decisions because a higher frequency of attacks indicates a severe asthma subtype associated with higher risk of death [49]. We observed an increased odds of uncontrolled asthma associated with the G-allele of *CC16* rs12270961 after adjusting for several confounders. No association was found with the exacerbation outcome. The rs12270961 polymorphism is located within an intron between exon 1 and 2, downstream of rs3741240 within the *CC16* (SCGB1A1) gene. The clinical significance of the function of this polymorphism remains unknown, and further investigations may be necessary.

To the best of our knowledge, the relationship between rs3741240 and asthma control has not been previously investigated. Pre-bronchodilator percent predicted forced expiratory volume in 1 s (FEV_1_) is included in the score to define uncontrolled asthma. The rs3741240 risk allele has been inconsistently linked to lung function in children [26, 62]. Furthermore, rs3741240 is associated with lower levels of protein in serum overall and in those with asthma, [9, 22] and low CC16 levels (serum) predict lower lung function [11, 21]. While people with asthma may have lower levels of CC16 serum or bronchoalveolar lavage (BAL) [63, 64], lung function does not always correlate with asthma control or exacerbations, especially when only evaluated at a single time point [65]. We do not know if the CC16 serum levels of the ALHS adult farmers with asthma varied by rs3741240 genotype and if those levels may be more informative than the polymorphisms alone. Therefore, CC16 may be involved in pathways related to lung function but relevance to asthma control or exacerbations is not known and warrants further study. Lastly, we did not observe any association between *CC16* polymorphisms with respiratory symptoms (wheeze, chronic bronchitis).

Our study had several notable strengths. Primarily, it had the largest sample size to date to investigate genetic associations between *CC16* polymorphisms and asthma in adults. Current asthma is well defined using a series of questions to classify current asthma, undiagnosed asthma, and asthma-COPD overlap – making it unlikely asthma cases were missing or misclassified. However, in a population of older adults with asthma, confusion with COPD remains possible. We also contributed to the existing knowledge base by being the first to evaluate *CC16* genetic associations with asthma subtypes and asthma control, including exacerbations using validated measures. In addition, there are limitations of the study. Our findings are limited to those with European ancestry; thus these findings may not be generalizable to other ancestries. CC16 levels were not measured in this study, thus we could not explore relationships between levels, genotypes, and asthma outcomes. Asthma control questions were only asked of individuals who reported a diagnosis of asthma; consequently, the 26% of individuals included as possible asthmatics based on symptoms and medication use did not have these data. Asthma control and exacerbation history can change over time so future studies may consider longitudinally measuring these outcomes.

## Conclusion

This study did not observe an association of *CC16* rs3741240 with asthma, asthma subtypes, asthma control, exacerbations or respiratory symptoms in adults from the ALHS. However, there was an association between the G-allele of *CC16* rs12270961 and uncontrolled asthma, a finding that requires further confirmation. The next steps may include evaluating *CC16* polymorphisms and asthma between farm-exposed and non-farm populations. Also, genetic studies may investigate potential mechanisms of rs12270961 related to asthma symptoms or lung function.

## Electronic supplementary material

Below is the link to the electronic supplementary material.

Supplementary Material 1

## Data Availability

Data are not public but may be available upon request to Stephanie London PI of the ALHS at the National Instutue of Environmental Health Sciences (NIEHS).

## List of abbreviations

AHS: Agricultural Health Study
ALHS: Agricultural Lung Health Study
ACQ: Asthma Control Questionnaire
BAL: Bronchoalveolar lavage
COPD: Chronic obstructive pulmonary disease)
CC16: Club cell secretory protein
FEV_1_: Forced expiratory volume in 1 s
FeNO: Fractional exhaled nitric oxide
GWAS: Genome-wide association study
GINA: Global Initiative for Asthma
HWE: Hardy-Weinberg Equilibrium
IgE: Immunoglobulin E
SCGB1A1: Secretoglobin family 1 A member 1 referred to in main text as CC16 gene
