# Correction to: CC16 polymorphisms in asthma, asthma subtypes, and asthma control in adults from the Agricultural Lung Health Study

**DOI:** 10.1186/s12931-022-02261-w

**Published:** 2022-12-16

**Authors:** K. C. Gribben, A. B. Wyss, J. A. Poole, P. A. Farazi, C. Wichman, M. Richards-Barber, L. E. Beane Freeman, P. K. Henneberger, D. M. Umbach, S. J. London, T. D. LeVan

**Affiliations:** 1 Department of Epidemiology, University of Nebraska Medical Center, Omaha, NE 68198 USA; 2 Epidemiology Branch, Department of Health and Human Services, National Institute of Environmental Health Sciences, National Institutes of Health, Research Triangle Park, NC USA; 3 Department of Internal Medicine, Division of Allergy and Immunology, University of Nebraska Medical Center, Omaha, NE 68198 USA; 4 Department of Biostatistics, University of Nebraska Medical Center, Omaha, NE 68198 USA; 5 Westat, Durham, NC USA; 6 Biostatistics and Computational Biology Branch, Department of Health and Human Services, National Institute of Environmental Health Sciences, National Institutes of Health, Research Triangle Park, NC USA; 7 Occupational and Environmental Epidemiology Branch, National Cancer Institute, Bethesda, MD USA; 8 Respiratory Health Division, National Institute for Occupational Safety and Health, Centers for Disease Control and Prevention, Morgantown, WV USA; 9 Department of Internal Medicine, Division of Pulmonary, Critical Care and Sleep, University of Nebraska Medical Center, Omaha, NE 68198 USA

**Correction to: Respiratory Research (2022) 23:305** **https://doi.org/10.1186/s12931-022-02211-6**

Following publication of the original article [1], the authors identified that the first author’s name was repeated twice in the authorship as last author. It has been corrected in this correction.

The original article has been corrected.
