# Supplementary material for: CC16 polymorphisms in asthma, asthma subtypes, and asthma control in adults from the Agricultural Lung Health Study
Source: Respir Res. 2022 Nov 9;23:305. doi: 10.1186/s12931-022-02211-6 (PMC9644514; doi:10.1186/s12931-022-02211-6)
Supplement: Supplementary file 1 — Supplementary Material 1 [file 12931_2022_2211_MOESM1_ESM.docx]

Supplement

**Table S1** *CC16* rs3741240 Genotype Pairwise comparisons for Asthma, asthma subtypes in the ALHS

|  | **CC16 rs3741240 genotype** | | | Linear Trend^‡^ |
| --- | --- | --- | --- | --- |
| **Outcome** | **AA** | **AG** | **GG** | *P*-value  Odds Ratio (95% CI) |
| **Asthma** |  |  |  | 0.53 |
| No (ref) | 249 | 843 | 834 |  |
| Yes | n = 133 | n = 501 | n = 486 |  |
| Odds Ratio (95% CI) | 0.9 (0.7–1.2) | 1.0 (0.9–1.2) | **1.0** | 1.0 (0.9–1.1) |
| *P*-value | 0.40 | 0.40 |  |  |
| **Atopy/Asthma*** |  |  |  | 0.78 |
| No/No (ref) | n = 209 | n = 718 | n = 706 |  |
| Yes/No | n = 33 | n = 100 | n = 111 |  |
| Odds Ratio (95% CI) | 1.0 (0.6–1.5) | 0.9 (0.7–1.2) | **1.0** | 1.0 (0.8–1.2) |
| *P*-value | 0.82 | 0.46 |  |  |
| No/Yes | n = 102 | n = 352 | n = 341 |  |
| Odds Ratio (95% CI) | 1.0 (0.8–1.3) | 1.0 (0.8–1.2) | **1.0** | 1.0 (0.9–1.1) |
| *P*-value | 0.94 | 0.94 |  |  |
| Yes/Yes | n = 30 | n = 137 | n = 132 |  |
| Odds Ratio (95% CI) | 0.8 (0.5–1.2) | 1.0 (0.8–1.3) | **1.0** | 0.9 (0.8–1.1) |
| *P*-value | 0.15 | 0.24 |  |  |
| **Eosinophilic/Asthma**^†^ |  |  |  | 0.96 |
| No/No (ref) | n = 229 | n = 765 | n = 781 |  |
| Yes/No | n = 4 | n = 14 | n = 10 |  |
| Odds Ratio (95% CI) | 1.3 (0.4-4.0) | 1.3 (0.5–2.9) | **1.0** | 1.2 (0.7-2.0) |
| *P*-value | 0.85 | 0.77 |  |  |
| No/Yes | n = 117 | n = 438 | n = 416 |  |
| Odds Ratio (95% CI) | 1.0 (0.7–1.2) | 1.1 (0.9–1.3) | **1.0** | 1.0 (0.9–1.1) |
| *P*-value | 0.53 | 0.28 |  |  |
| Yes/Yes | n = 11 | n = 26 | n = 31 |  |
| Odds Ratio (95% CI) | 1.2 (0.6–2.4) | 0.9 (0.5–1.5) | **1.0** | 1.0 (0.7–1.5) |
| *P*-value | 0.45 | 0.36 |  |  |

Genotypes entered regression models as a 3-level categorical variable (AA, AG, GG (ref)) .

Sample sizes (n) are the number of individuals for each genotype-outcome group combination.

Multivariable logistic regression model adjusted for gender (Male or Female), state (NC,IA), age (continuous), smoking status (current, former, never) were used to estimate odds ratios and 95% confidence intervals. Multinomial logistic regression models to estimate adjusted odds ratios and 95% confidence intervals for asthma subtypes. Adjusted for gender, state (NC, IA), age, smoking status (current, former, never). Models were not adjusted for race because sample population is 99.9% white.

*Atopy/asthma = atopy defined as 1 or more allergen-specific IgE ≥ 0.70 IU/mL cross-classified with self-reported asthma (yes/no) resulting in four groups.

†Eosinophilia/asthma = eosinophilic defined by fractional exhaled nitric oxide (FeNo) > 50 ppb cross-classified with self-reported asthma (yes/no) resulting in four groups.

‡Linear trend ORs (additive genetic effect) (results also shown in Table 2) represents a multiplicative change in odds per each additional copy of allele A.

**Table S2**. Associations between *CC16* rs3741240 and chronic bronchitis and wheeze stratified by asthma

|  | **Asthma (N = 1120)** | | | | | **No Asthma (N = 1926)** | | | | |
| --- | --- | --- | --- | --- | --- | --- | --- | --- | --- | --- |
|  | **rs3741240 Genotype**  **n (row %)** | | |  |  | **rs3741240 Genotype**  **n (row %)** | | |  |  |
| **Outcome** | **AA** | **AG** | **GG** | **Adj OR**  **(95% CI)*** | **p** | **AA** | **AG** | **GG** | **Adj OR**  **(95% CI)*** | **p** |
| **Chronic Bronchitis*** |  |  |  |  | 0.80 |  |  |  |  | 0.77 |
| No | 110 (12.4) | 393 (44.2) | 386 (43.4) | **Ref** |  | 235 (12.9) | 800 (43.9) | 788 (43.2) | **Ref** |  |
| Yes | 22 (10.3) | 99 (46.5) | 92 (43.2) | 1.0 (0.8,1.2) |  | 12 (16.9) | 27 (38.0) | 32 (45.1) | 1.1 (0.7,1.5) |  |
| **Wheeze**^†^ |  |  |  |  | 0.47 |  |  |  |  | 0.32 |
| No | 44 (13.3) | 131 (39.7) | 155 (47.0) | **Ref** |  | 216 (13.0) | 720 (43.2) | 730 (43.8) | **Ref** |  |
| Yes | 88 (11.4) | 361 (46.8) | 323 (41.8) | 1.1 (0.9,1.3) |  | 31 (13.7) | 106 (46.9) | 89 (39.4) | 1.1 (0.9, 1.4) |  |

Additive genetic model for the rs3741240 genotype coded as the number of copies of the A allele; thus ORs reflect a multiplicative change in the odds per additional copy of the A allele.

Multivariable logistic regression model adjusted for gender, state, smoking status, and age.

*Chronic bronchitis defined as individuals with both chronic cough and phlegm. Chronic cough defined as having a cough on most days, for three consecutive months or more during the year for a duration of two years or more. Similarly, chronic phlegm will be defined as bringing up phlegm on most days for three consecutive months or more during the year, for a duration of two years or more.

†Wheeze defined as having wheezing or whistling in your chest during the past 12 months or wheeze when exerting during the past 12 months.

**Table S3.** Associations between *CC16* rs12270961 and chronic bronchitis and wheeze stratified by asthma

|  | **Asthma (N = 1120)** | | | | | **No Asthma (N = 1926)** | | | | |
| --- | --- | --- | --- | --- | --- | --- | --- | --- | --- | --- |
|  | **rs12270961 Genotype**  **n (row %)** | | |  |  | **rs12270961 Genotype**  **n (row %)** | | |  |  |
| **Outcome** | **AA** | **AG** | **GG** | **Adj OR**  **(95% CI)*** | **p** | **AA** | **AG** | **GG** | **Adj OR**  **(95% CI)*** | **p** |
| **Chronic Bronchitis*** |  |  |  |  | 0.49 |  |  |  |  | 0.46 |
| No | 37 (4.2) | 295 (33.2) | 557 (62.7) | **Ref** |  | 71 (3.9) | 604 (33.1) | 1148 (63.0) | **Ref** |  |
| Yes | 7 (3.3) | 68 (31.9) | 138 (64.8) | 1.1 (0.8,1.4) |  | 5 (7.0) | 23 (32.4) | 43 (60.6) | 0.9 (0.6–1.3) |  |
| **Wheeze**^†^ |  |  |  |  | 0.97 |  |  |  |  | 0.32 |
| No | 12 (3.6) | 111 (33.6) | 207 (62.7) | **Ref** |  | 67 (4.0) | 541 (32.5) | 1058 (63.5) | **Ref** |  |
| Yes | 32 (4.2) | 252 (32.6) | 488 (63.2) | 1.0 (0.8–1.3) |  | 9 (4.0) | 85 (37.6) | 132 (58.4) | 0.9 (0.7–1.1) |  |

Additive genetic model: genotypes coded as having 0, 1, or 2 copies of the G allele for rs12270961. OR represents the multiplicative change in odds per additional G allele. Multivariable logistic regression model adjusted for gender, state, smoking status, and age.

*Chronic bronchitis defined as individuals with both chronic cough and phlegm. Chronic cough defined as having a cough on most days, for three consecutive months or more during the year for a duration of two years or more. Similarly, chronic phlegm will be defined as bringing up phlegm on most days for three consecutive months or more during the year, for a duration of two years or more.

†Wheeze defined as having wheezing or whistling in your chest during the past 12 months or wheeze when exerting during the past 12 months

| **Table S4**. Associations of CC16 polymorphisms with atopic asthma in the ALHS (N = 3046) | | | | | | | | | | |
| --- | --- | --- | --- | --- | --- | --- | --- | --- | --- | --- |
|  | **CC16 rs12270961** | | | | | **CC16 rs3741240** | | | | |
|  | **Genotype n (row %)** | | |  |  | **Genotype n (row %)** | | |  |  |
|  | **AA** | **AG** | **GG** | **Adj OR**  **(95% CI)*** | **p** | **AA** | **AG** | **GG** | **Adj OR**  **(95% CI)*** | **p** |
| **Atopy/Asthma^*^** |  |  |  |  | 0.65 |  |  |  |  | 0.82 |
| No/No | 48 (4.2) | 356 (31.4) | 729 (64.3) | **Ref** |  | 143 (12.6) | 492 (43.4) | 498 (44.0) | **Ref** |  |
| Yes/No | 29 (3.9) | 260 (35.0) | 455 (61.2) | 0.9 (0.8, 1.1) |  | 99 (13.3) | 326 (43.8) | 319 (42.9) | 1.0 (0.9, 1.2) |  |
| No/Yes | 25 (4.4) | 186 (32.7) | 358 (62.9) | 0.9 (0.8, 1.1) |  | 76 (13.4) | 249 (43.8) | 244 (42.9) | 1.0 (0.9, 1.2) |  |
| Yes/Yes | 18 (3.4) | 173 (33.0) | 334 (63.6) | 1.0 (0.8, 1.2) |  | 56 (10.7) | 240 (45.7) | 229 (43.6) | 1.0 (0.8, 1.1) |  |

Additive genetic model for the rs12270961 genotype coded as the number of copies of the G allele; for the rs3741240 genotype, as the number of copies of the A allele; thus, ORs reflect a multiplicative change in odds per additional copy of the designated allele.

Multinomial logistic regression model to estimate adjusted odds ratios and 95% confidence intervals for asthma subtypes. Adjusted for gender, state (NC, IA), age, smoking status (current, former, never). Models were not adjusted for race because sample population is 99.9% white.

*Atopy/asthma = atopy defined as 1 or more allergen-specific IgE ≥ 0.35IU/mL cross-classified with self-reported asthma (yes/no) resulting in four groups.
